# Effects of a Brief Music–Movement Active Break on Executive Functions, Mathematics, and Engagement in Preschool Children

**DOI:** 10.3390/children13070855

**Published:** 2026-06-26

**Authors:** Dimitris Chatzopoulos, Eleni Mouchou-Moutzouridou, Elpida Pogonidou, Loukia Kapodistria

**Affiliations:** School of Physical Education and Sport Science, Aristotle University of Thessaloniki, 57001 Thessaloniki, Greece; mouchoue@phed.auth.gr (E.M.-M.); epagonic@phed.auth.gr (E.P.); kapodist@phed.auth.gr (L.K.)

**Keywords:** dance, physical activity, cognitive function, involvement, enjoyment

## Abstract

**Highlights:**

**What are the main findings?**

- The music–movement group performed significantly better in inhibitory control compared to the control group.

**What are the implications of the main findings?**

- A short music–movement active break may improve preschool children’s inhibitory control.
- It may also enhance children’s engagement in classroom activities.

**Abstract:**

**Background**: Active breaks are short periods of physical activity integrated into classroom. The aim of this study was to examine the effects of a brief music–movement active break on preschool children’s executive functions, mathematics performance, lesson enjoyment and engagement in classroom activities. **Methods**: The sample consisted of 53 preschool children (five- to six-year-old) who were randomly assigned to an intervention group (*n* = 25) and a control (*n* = 28). The intervention group participated in a 5-min music–movement active break, whereas the control group continued with regular seated classroom activities. At baseline and post-intervention, children completed measures of executive functions (inhibitory control, working memory, and cognitive flexibility), mathematics assessments and engagement. At the end of the intervention children also reported their enjoyment using a 3-point smiley-face scale. **Results**: At post-intervention, the music–movement group demonstrated significantly better performance in inhibitory control compared to the control. Moreover, the intervention group exhibited higher levels of engagement in classroom activities compared to baseline. No significant group differences were observed in mathematics outcomes and self-reported enjoyment, as assessed by the smiley-face scale. **Conclusions**: The findings suggest that a brief music–movement active break can positively influence children’s inhibitory control and may also contribute to improved task engagement. Although smiley-face scales are commonly used in preschool research, classroom observations from the present study suggest potential limitations in their suitability for assessing enjoyment in this age group.

## 1. Introduction

Executive functions (EF) refer to a set of cognitive processes that are essential for children’s cognitive and social development [1]. According to Diamond [2], three core components of EF can be identified: (a) working memory, defined as the ability to retain and manipulate information; (b) inhibition, the ability to maintain focus on a task by resisting impulses and distractions; and (c) cognitive flexibility, the capacity to think quickly and adaptively in order to solve problems. EF play a key role in helping young children process and acquire new information and have been linked to academic skills such as mathematics and language development [3,4,5].

The association between physical activity and cognitive performance is supported by evidence indicating that motor and cognitive activities activate overlapping brain regions [6]. Currently, classroom activity breaks (AB) (short periods of physical activity performed inside the classroom as a pause from the lesson activities), are receiving considerable attention in the field of education, particularly for their potential to enhance cognitive performance and on-task behavior [7,8,9]. However, the literature examining the effects of a single session of exercise on the cognitive functions of preschool children has produced mixed findings [10,11]. Some studies suggest that AB can improve executive function outcomes in preschool children [9,12]. For example, Palmer et al. [13] used a 30-min movement program incorporating a variety of activities (e.g., hopping between targets, throwing, and dribbling) and found improvements in sustained attention. Likewise, Zhang et al. [12] reported enhanced cognitive flexibility following a 25-min physical activity session that included exercises such as squats, walking, hopping, jumping, and relay races. Moreover, Du et al. [9] also observed improvements in working memory and inhibitory control after 20 min of physical activity. In contrast, other studies have reported no improvements in EF following physical activity [14,15]. For example, Haas et al. [14] implemented a 20-min motor coordination intervention and found no differences in EF or enjoyment. Similarly, Stein et al. [15] employed a 20-min coordinative intervention program (throwing or kicking balls at targets using both sides of the body) and reported no significant effects on EF. However, some evidence indicates that sedentary activities may be more beneficial compared to AB [16]. For example, Tandon et al. [16] implemented a 15-min aerobic activity session (chasing games, running, and jumping), and reported better inhibitory control in the sedentary group engaged in activities such as storytelling, crafts, and coloring. The authors suggested that following physically active periods, younger children may exhibit residual overactive behavioral responses, which could lead to increased errors in inhibitory tasks.

A common characteristic of the above studies is the long duration of the AB, typically exceeding 15 min. However, AB of such duration have limitations that restrict their practical implementation in real classroom settings [17]. Teachers have indicated a preference for shorter active breaks (≤5 min) in order to minimize time away from academic instruction [17,18,19]. Indeed, when we proposed the implementation of a 20-min AB within the classroom, teachers responded negatively, arguing that such a duration resembles a physical education lesson rather than a brief classroom AB. A review of the literature on ≤5-min AΒ supports the feasibility and cognitive benefits of very short interventions, although existing evidence is limited to children aged 9–12 years [19,20]. To the best of our knowledge, no corresponding study has examined the effects of ≤5 min AB in preschool-aged children. Therefore, the present study aimed to investigate the effects of a short, 5-min AΒ, an approach that was also supported by preschool teachers.

Another limitation of previous studies is the type of physical activity used in AB (e.g., jumping jacks, marching, jumping, and running in place). In a pilot study in which we implemented such activities, children showed limited interest in participating. Moreover, activities such as relay races, chasing games, and ball skills (e.g., throwing or kicking a ball), which have also been used in previous research [15,16], were not included due to safety concerns associated with implementing such activities within the classroom environment. For these reasons, preschool teachers suggested incorporating music–movement activities, which are particularly popular among young children. Moving in synchrony with the rhythm of music has been shown to positively influence affective arousal and enjoyment [21]. Previous studies examining repeated, long-term AB (i.e., chronic AB, performed multiple times per week over several weeks or months) have reported positive effects of dance-based activity breaks on executive functions [4,22]. However, to the best of our knowledge, no study has investigated the effects of a single short music–movement programs (≤5 min) on executive functions in preschool children.

Engagement refers to the degree to which a child is focused, attentive, and actively engaged in classroom activities [23]. Engagement in learning activities (i.e., classroom behaviors such as attentiveness and adherence to instructions) has been shown to predict academic achievement [24,25]. Physical activity has been positively associated with on-task behavior in classroom settings among elementary school children [26,27,28]. Similar positive findings have been reported in the limited number of studies conducted in preschool populations. Specifically, Webster et al. [29] reported positive effects of AΒ consisting of gross motor movements (e.g., marching, bunny hops, scissor kicks, and lunges) on on-task behavior in preschool-aged children. However, no study has examined the effects of a music–movement program on children’s engagement. Music plays an important role in calming young children, as it positively influences their emotional and psychological state [30]. Therefore, the hypothesis of the present study was that a music–movement program would have a positive effect on children’s engagement in learning activities.

Long bouts of AB (>15 min) have been shown to improve EF outcomes and engagement in preschool children [9,27]. However, shorter bouts (≤5 min), which have demonstrated positive results in elementary-aged children [19,20], may be more practical for implementation in real classroom settings. Given the important role of executive functions in early learning and development, the primary focus of the present study was to examine the effects of a brief music–movement active break on executive functions, while mathematics performance, engagement, and enjoyment were examined as secondary outcomes.

## 2. Materials and Methods

### 2.1. Participants

The sample size was calculated using G*Power version 3.1.9.2 [31], by setting f = 0.25 and power 0.90, the required sample size was 46. Moreover, a dropout rate of 10% was considered; therefore, the minimum sample size should have consisted of 50 participants [32]. The inclusion criterion for participation was the absence of any physical or mental disorders among the children, as reported by their teacher. The final sample included 53 children from four kindergartens in the same district (5–6 years old). Using a computer-generated random number sequence created in Microsoft Excel, the four kindergartens were randomly allocated to either the intervention group (two kindergartens; *n* = 25, 14 boys and 11 girls; age = 64.32 ± 5.87 months) or the control group (two kindergartens; *n* = 28, 15 boys and 13 girls; age = 64.28 ± 5.85 months). Randomization was conducted at the kindergarten level by an independent researcher who was not involved in any study procedures. Thus, this study was a cluster-randomized trial, with kindergartens serving as the unit of randomization and two clusters allocated to each study arm. The research was conducted in accordance with the ethical guidelines of the local University. Informed consent was obtained from the guardian of the children, which also gave their oral assent.

### 2.2. Procedures

**Figure 1:**
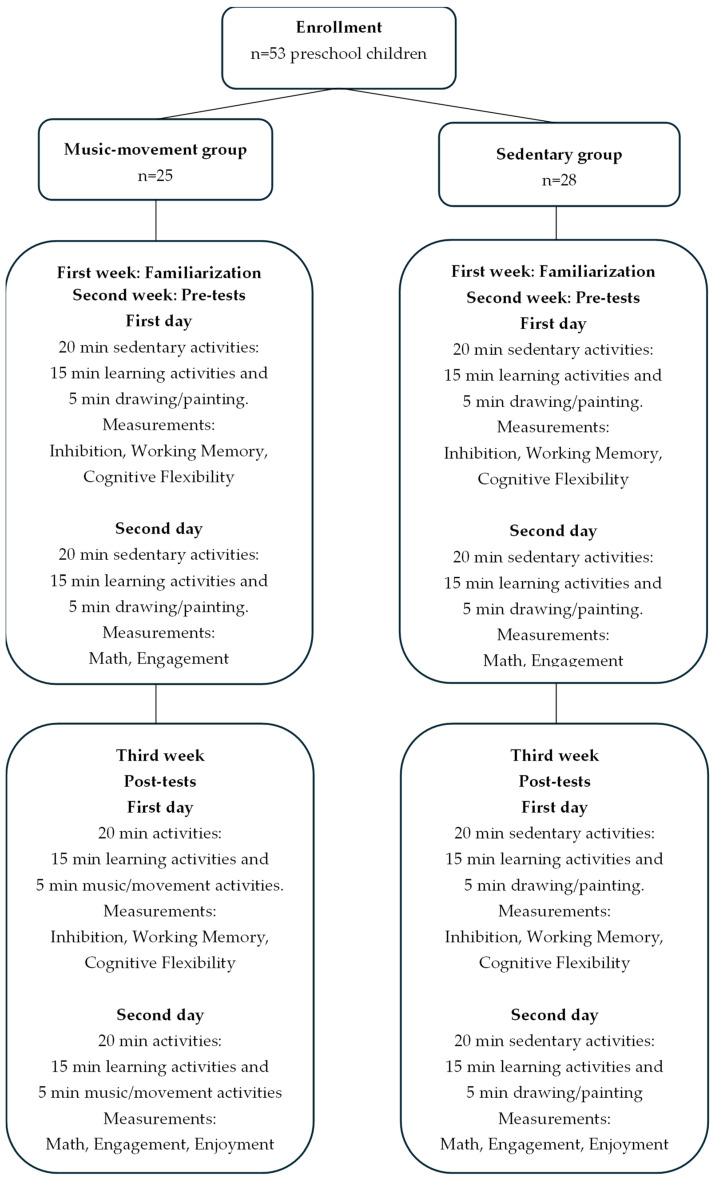
Flow diagram of the intervention and the control condition.

During the first week, children were familiarized with the researchers and the testing procedures (Figure 1). The research team consisted of six trained assistants who were not blinded to group allocation. In addition, the teachers were aware of group assignment. Blinding teachers was not feasible because each preschool class was instructed by its regular classroom teacher. Replacing teachers with researchers could have disrupted the children’s familiar learning environment and altered their behavior during the intervention, thereby reducing the ecological validity of the study. Consequently, the possibility of observer or expectancy bias cannot be entirely excluded. During the familiarization phase, the classroom teacher introduced the research assistants to the children, and they engaged in play-based activities to establish rapport. The same experimenters were subsequently assigned to the same children for all measurement procedures to ensure consistency across assessments.

In the second week, pre-tests were administered to both the intervention and control group approximately 20 min after the onset of the lesson. In the third week, post-tests were conducted for both groups at the same time point (i.e., midway through the lesson, approximately 20 min after its onset). Specifically, in the intervention group, children participated in 15 min of structured learning activities, followed by a 5-min music–movement activity (active break), after which the post-tests were administered. In the control (sedentary) group, children completed 15 min of structured learning activities, followed by 5 min of sedentary activities (drawing and painting), and then the post-tests were administered (Figure 1). All research procedures were conducted between 10:00–12:00 a.m. Drawing/painting was selected as the control condition because it is a common and engaging sedentary classroom activity for preschool children and has been widely used in previous studies comparing active breaks with sedentary activities [12,13,16].

To reduce potential fatigue effects, pre- and post-assessments were administered over two days in a fixed order. On the first day, EF measures were administered, including inhibition, working memory, and cognitive flexibility. On the second day, mathematics assessments were administered, along with measures of children’s engagement and enjoyment during the activities. On both testing days, children first participated in the activity assigned to their group and subsequently completed the post-test assessments. Therefore, both the music–movement active break and the seated break activity (drawing/painting) were administered on each testing day, with post-test assessments conducted afterward (Figure 1). The present research design was selected in consideration of the limited attention span of preschool children [33]. According to the official timetable of the Greek Ministry of Education, the duration of preschool organized activities is 40–45 min. However, due to young children’s restricted attentional capacity, a typical preschool organized activity (circle-time session) generally lasts 15–20 min and focuses on a single whole-group activity, such as vocabulary development, early writing, reading, or mathematics concepts [34]. At the end of the circle time, it is common practice in early childhood classrooms to engage children in a pleasant and less demanding cognitive activity (e.g., drawing or completing a puzzle) as a brief classroom break before the transition to the next learning activity [24,35]. The aim of the present study was to examine the effects of a music–movement active break on preschool children’s executive functions, mathematics performance, and engagement, compared with a drawing break (sedentary control group). To maintain ecological validity and reflect typical classroom practice, both the intervention and control groups participated in 15-min circle-time lessons followed by a 5-min break activity (music–movement active break or drawing/painting). Consequently, on both testing days, pre- and post-test assessments were administered approximately 20 min after the beginning of the lesson in both groups (Figure 1). This research design enables the investigation of both between-group differences and within-group (pre–post) changes in the examined variables.

The specific sequence of EF tasks (inhibition—working memory—cognitive flexibility) was selected to accommodate the limited attention span of preschool children. This fixed order accounts for the mental energy required for each task and reflects a progression from lower to higher cognitive demands. The battery begins with the Flanker task (inhibitory control), a fast-paced, gamified activity (‘feeding the fish’) that serves as an engaging ‘hook’. Requiring minimal strategic thinking, it acts as an effective cognitive warm-up. This is followed by the Corsi Block task (working memory), a visuospatial measure that, while more demanding than the Flanker, remains intuitive. It effectively bridges the gap between a reaction-time task (Flanker task) and a complex planning task (Tower of London). The sequence concludes with the Tower of London (cognitive flexibility), the most mentally taxing task in the battery, requiring multi-step planning and rule-following. Positioning this high-load task last, minimizes potential frustration or disengagement and ensures that performance on simpler tasks is not negatively affected by cognitive overload.

### 2.3. Measurements

#### 2.3.1. Inhibitory Control

The assessment of inhibitory control was conducted using a computerized Flanker task with fish stimuli, delivered via Psytoolkit software version 3.7.0 [36,37]. The task included two experimental conditions: congruent and incongruent trials [38,39]. Participants were required to indicate the direction of the central target fish while ignoring the surrounding flanker fish. To enhance engagement, the task was presented in a game-like format in which participants “fed” the hungry central fish [20]. Statistical analysis was based on mean reaction time (RT) for correct responses, with higher RT values reflecting lower attentional control. A second dependent variable was the percentage (%) of correct answers. Test reliability was evaluated using a test–retest procedure with 13 children over a 3-day interval, yielding satisfactory results (ICC > 0.67). Reliability estimates were derived from a separate sample of children who did not participate in the main study.

#### 2.3.2. Working Memory 

The Corsi block-tapping test (Corsi 1972) was administered via Psytoolkit [37]. The interface presented nine blue cubes arranged randomly on a black background. During each trial, the cubes briefly changed color from blue to yellow in specific sequences. Participants were instructed to reproduce each sequence by pointing to the cubes in the same order in which they appeared. Performance was assessed based on the longest sequence of blocks the participant could correctly recall. Test–retest reliability was evaluated using a separate sample of 12 children over a two-day interval, yielding a high intraclass correlation coefficient (ICC = 0.88). Similar reliability levels have been reported in previous research, with values ranging from 0.81 to 0.89 among children aged 3 to 6 years [40].

#### 2.3.3. Cognitive Flexibility

The Tower of London task (ToL) involves a wooden board with three pegs of different heights and three colored cylinders [41]. Children are asked to rearrange the cylinders on the pegs to match a given target configuration while following specific rules (e.g., moving only one cylinder at a time and completing the task within a set number of moves). The procedure includes two practice trials followed by ten test trials, with task difficulty increasing progressively. The highest score is 10. In a pilot study using a separate sample of 13 children (test–retest method), the task demonstrated acceptable reliability, with an intraclass correlation coefficient (ICC) of 0.73.

#### 2.3.4. Math Outcome Measures

The math assessments were adapted from Mavilidi et al. [42] and included (a) number line estimation and (b) numerical magnitude comparison tasks:

- Number line estimation: Children were asked to identify the positions of the numbers 2, 3, 5, 7, and 9 on a number line that only displayed the numbers 1 at the start and 10 at the end, with all other numbers omitted. They earned 1 point for each correct placement, with a maximum possible score of 5.
- Numerical magnitude comparison: Children were asked to identify the larger number in pairs of digits (e.g., 2 vs. 5, 4 vs. 9, 5 vs. 8, 4 vs. 7, 6 vs. 9). They received 1 point for each correct response, with a maximum possible score of 5.

#### 2.3.5. Engagement

Two trained data collectors conducted classroom observations using the engagement/involvement dimension of the Child Observation in Preschool (COP) protocol [23,43]. The engagement dimension measures the extent to which children are focused and actively engaged in classroom activities. Observations are scored on a five-point scale: a rating of 1 (low involvement) indicates a lack of interest or being off-task (e.g., drumming with a pencil), while a 5 (high involvement) reflects intense, sustained concentration. Moderate involvement refers to the behavior of a child who is focused on an activity, briefly interrupts it (e.g., by looking elsewhere), and then returns to it again.

The engagement assessment instrument is resource-intensive and requires extensive observer training prior to data collection. Given that only two trained observers were available and following the instrument’s protocol recommending six children per observer per classroom, a sub-sample of 24 children was selected. Specifically, 12 children from the intervention group (six per classroom) and 12 from the control group (six per classroom) were chosen via stratified random sampling. The criterion for selection was to ensure a balanced distribution of boys and girls across both study conditions. Children’s behavior was coded using repeated momentary observations, with each coding decision recorded at approximately 8–10-s intervals. Each target child was observed 10 times (total observation duration approximately 8–10 min). Inter-observer agreement reached 91%, a level comparable to that reported by Miranda et al. [23].

#### 2.3.6. Enjoyment

At the conclusion of the intervention, children were asked to indicate their enjoyment of the lesson by responding to the question, “Did you enjoy the lesson today?”, using a 3-point faces scale (happy, neutral, and sad faces), ranging from 1 (“I did not like it at all”) to 3 (“I liked it a lot”) [14].

### 2.4. Intervention

The intervention group carried out the same learning activities as the control group for 15 min and then followed a music-and-movement program for 5 min. The choreography of the intervention group was demonstrated by a physical education teacher with dance qualification, and children were asked to mimic the movements. The dance instructor was not involved in either the assessment procedures or the collection of study data. The title of the song in the intervention group was “Dance Monkey”, by Tones and I (producer Konstantin Kersting), and the choreography was based on basic movement steps (e.g., marching, jumping, step side). Moreover, locomotor dance movements were combined with expressive arm gestures to help the children convey the song’s lyrics (e.g., “cry” and “happy”). For example, in the lyrics “Ooh, I see you, see you, see you every time. And oh my, I like your style. You, you make me, make me, make me wanna cry,” the children used arm movements to represent the ideas of “I see you” and “cry.” The choreography used in the intervention was adapted from the YouTube video “Dance Monkey” Zumba Kids Choreography https://www.youtube.com/watch?v=GRM9h8EQ6Bw (accessed on 1 June 2026). The song Dance Monkey has a tempo of approximately 98 beats per minute (BPM) and has a slightly “bouncy” and syncopated feel, making it ideal for music–movement activities. Moreover, some beats are emphasized off the main rhythm, creating a playful pattern that further motivates children to engage in movement.

For the cool-down phase and the gradual return of the children to their seats, the following movements were performed in synchrony with the music (Calm Down Song for Kids, https://www.youtube.com/watch?v=TRLS-6s3yfQ (accessed on 1 June 2026):

- Standing upright with their arms by their sides, children stretched (raised) their arms overhead and then lowered them back alongside the body.
- They then bent forward to touch their toes and returned to an upright position with their arms by their sides.
- Finally, they slowly sat down in their seats and took a calm, deep breath.

The seated group (control) followed for 15 min the regular lesson activities on the same topic, just like the intervention group, and then engaged in 5 min drawing/painting. Following the 15 min of lesson activities and the 5 min of drawing/painting, children performed the pre- and post-tests (i.e., approximately 20 min after the onset of the lesson).

### 2.5. Statistical Analyses

Separate analyses of covariance (ANCOVA) were performed to examine differences between the intervention and control group. The post-test scores were the dependent variables and the corresponding pretest scores were the covariates. The paired samples *t*-test was used to examine within-group differences between pre- and post-test. Furthermore, effect sizes were reported using partial eta squared (ηp^2^) for ANCOVA and Cohen’s d for paired-samples *t*-tests. Reliability was assessed using a two-way, random-effects, single measure Intraclass Correlation Coefficient (ICC). All statistical analyses were carried out employing SPSS (version 28). Statistical significance was set at *p* ≤ 0.05.

## 3. Results

Mean and standard deviation of the dependent variables are presented in Table 1.

### 3.1. Inhibitory Control (Flanker Task)

ANCOVA revealed that children in the intervention group exhibited significantly faster reaction times compared to those in the control group (F = 6.18, *p* = 0.016, ηp^2^ = 0.11). Furthermore, the intervention group improved significantly from pre-test to post-test (t = 3.65, *p* = 0.001, Cohen’s d = 0.73). Regarding the percentage of correct answers (%), there was no significant difference between the two groups (F = 0.357, *p* = 0.55, ηp^2^ = 0.007).

### 3.2. Working Memory (Corsi Block Test)

There was no significant difference between the two groups (F = 0.108, *p* = 0.743, ηp^2^ = 0.002).

### 3.3. Cognitive Flexibility (Tower of London)

There was no significant difference between the two groups (F = 0.589, *p* = 0.446, ηp^2^ = 0.012).

### 3.4. Math Performance

Regarding number line estimation and numerical magnitude comparison, there were no significant differences between the two groups (F = 0.023, *p* = 0.88, ηp^2^ = 0.00, F = 0.176, *p* = 0.677, ηp^2^ = 0.004, respectively).

### 3.5. Engagement

ANCOVA revealed no significant difference between the intervention and control groups (F = 0.819, *p* = 0.376, ηp^2^ = 0.038). However, paired-samples *t*-test indicated a significant improvement from pre- to post-measurement in the intervention group (t = 2.24, *p* = 0.046, Cohen’s d = 0.648). In contrast, no significant change was observed in the control group between pre- and post-measurement (t = 1.00, *p* = 0.339, Cohen’s d = 0.289).

### 3.6. Enjoyment

There were no significant differences between the groups (t = 0.47, *p* = 0.63, d = 0.59).

## 4. Discussion

The aim of this study was to investigate the effects of a brief music–movement AB on EF, math performance, enjoyment and engagement of preschool children. According to the results of the study, the intervention group showed better inhibition control and higher levels of engagement after the music–movement session compared to the sedentary group. There were no significant differences regarding working memory, cognitive flexibility, math performance and enjoyment of the lesson.

Studies of elementary school children consistently show improvements in inhibition after a single session of active break [11,20,44]. However, the limited number of studies examining the effects of a single AB on preschool children’s inhibition are incongruent. Specifically, Ureña et al. [45] reported improved behavioral self-regulation after 15 min circuit with bike, and Palmer et al. [13] reported better ability to sustain attention, after 30 min of various motor skills (e.g., hopping from target to target, throwing, dribbling a ball), compared to sedentary. However, Russo et al. [33] reported no differences on selective attention between 20–30 min physical activity session and indoor activities (cutting, drawing, and coloring). Whereas Tandon et al. [16] reported better inhibitory control in the sedentary group (engaged in activities such as storytelling, crafts, and coloring) compared to a single session of aerobic activities. The authors proposed that after a short period of physical activity, preschool children might show lingering hyperactive behaviors, potentially resulting in more mistakes on tasks requiring inhibition. The study of Tandon et al. [16] contained a 15-min aerobic activity session with chasing games, running, and jumping. When children engage in such activities, they tend to go all out, reach very high heart rates and enter a heightened state of stimulation. This hyperarousal may explain why children in the study of Tandon et al. [16] showed reduced performance in inhibitory control compared to the sedentary group. In our study, children were required to concentrate and synchronize their movements to the beat of the song, which appeared to prevent them from reaching a heightened level of physiological arousal. The children remained focused on performing the music–movement activities, and at no point did they exhibit signs of breathlessness or sweating, indicating that they did not reach a high level of physical exertion or overstimulation. However, this observation should be interpreted with caution, as it is based on a subjective assessment rather than an objective measure of exercise intensity. Future research should incorporate objective measures of the intensity of music–movement activities, such as accelerometry or ratings of perceived exertion, to provide a more accurate evaluation and further clarify this issue. During the music–movement activities, children are not only required to synchronize their movements to the rhythm of the music but also to suppress their innate tendency to perform fast and high-intensity movements. This process demands a high degree of self-control and sustained attention, which may, in turn, transfer to subsequent learning activities in the classroom. However, further research is needed to substantiate this interpretation.

Regarding working memory and cognitive flexibility, the findings of the present study indicate no significant differences between the two groups. This lack of effect aligns with previous research reporting that a single bout of AB does not enhance working memory [46,47] or cognitive flexibility [12,48]. Conversely, other studies have demonstrated positive effects of active break interventions on both working memory [49,50] and cognitive flexibility [51,52]. These inconsistent findings may be attributed to procedural discrepancies, such as variations in the intensity, duration, or neuromuscular demands of physical activity. For instance, exercise intensities and durations vary widely across the literature, with heart rates ranging from 120 to 160 bpm and session durations spanning 10 to 50 min [48]. Each of these factors may differentially affect executive function outcomes. In the present study, physical activity intensity was not objectively assessed. Therefore, it is possible that the intensity of the music–movement break was insufficient to induce improvements in working memory and cognitive flexibility. To address this limitation, future research should investigate music–movement active breaks delivered at varying intensity levels.

In the present study, children demonstrated increased engagement levels during learning activities following the music–movement intervention relative to baseline levels. This finding appears to be consistent with the limited literature of classroom-based physical activity breaks on preschool children engagement [29,53]. Webster et al. [29] found that a short bout of active break involving gross motor activities, such as marching, bunny hops, scissor kicks, and lunges, had positive effects on on-task behavior among preschool-aged children (mean age ≈ 3.8 years). Similar positive results on on-task behavior after AB have been reported with early primary children, regardless their implementation time (morning or afternoon) [26]. Preschool children often struggle to sustain attention during prolonged sedentary activities, which can lead to restlessness and disengagement [33]. A distinctive feature of the present study is that it addresses this challenge by satisfying children’s natural need for movement and play through playful, rhythmic activities. Rhythmic movement has been shown to strengthen self-regulatory capacities by engaging neural systems responsible for managing behavior and attention [54,55]. The inherently enjoyable nature of music–movement activities also promotes active participation and sustained involvement, which can translate into improved focus when children return to academic tasks [55]. Therefore, by simultaneously supporting behavioral regulation and motivation, music–movement activities represent a promising approach for enhancing on-task behavior in preschool settings. However, given the limited number of studies in this area, further research is needed.

Dancing is among children’s most favored activities, and it is closely related to enjoyment [56,57]. On the other hand, enjoyment reduces anxiety, increases children’s motivation for learning and influences their academic performance in a positive way [58,59,60]. Therefore, it was expected that the music–movement group would present higher scores in enjoyment and engagement (on-task behavior) than the sedentary group. However, this hypothesis was partially confirmed. Although children showed greater engagement in the intervention group there was no significant difference regarding enjoyment. The findings of the present study is in accordance with Haas et al. [14] who reported no significant differences between AB and sedentary group. Similarly to our study, in the study of Haas et al. [14], preschool children answered on a 3-point ordinal scale with child-oriented graphics. In contrast, in the study of Mavilidi et al. [42], children showed higher interest ratings in the active learning conditions than in the sedentary condition. One possible explanation for the conflicting findings may be the difference in the Likert scale format (5-point vs. 3-point) used in the studies. In the study of Mavilidi et al. [42], children rated their enjoyment using a 5-point Likert scale with smiley faces, and it is well established that a 5-point Likert scale generally provides greater sensitivity and discrimination than a 3-point scale [61]. Therefore, the use of a 5-point scale in future studies may yield different results. However, some authors argue that preschool children (aged 3–5) are unlikely to use 5-point scales reliably, which is why a 3-point scale was selected in this study [62,63]. Clearly, further research is needed on this topic.

The findings related to enjoyment should be interpreted with caution. Although pictorial smiley-face scales are commonly used with preschool children because of their simplicity and developmental appropriateness [14,42], the use of a single-item 3-point scale may have limited sensitivity to detect subtle differences in affective responses. Moreover, during the assessment process, examiners observed that some children appeared to select the smiling-face responses without carefully considering the question. This tendency may have contributed to the very high enjoyment scores observed across both groups and resulted in a ceiling effect. Consequently, the absence of significant differences between groups may partly reflect limitations of the measurement tool rather than a true lack of differences in enjoyment. Future studies should consider employing more comprehensive and psychometrically validated measures of enjoyment for young children to better capture variations in their affective experiences.

One limitation of the study is that it was implemented as a class-level intervention involving different teachers for each class. In the preschool educational system, each class has its own teacher, and variations in teaching style, enthusiasm, and classroom management may have influenced the findings. Furthermore, because each study arm included only two kindergartens, teacher effects could not be isolated from intervention effects. Future studies should include a larger number of clusters and implement designs that better control for teacher-related influences. Another limitation of the study is related to the young age of the participants. Due to preschool children’s developmental immaturity, it cannot be certain that they consistently performed to the best of their ability during the assessments, which may have affected the result of the measurements. Third, due to the resource-intensive nature of the engagement observation protocol, this variable was assessed in a sub-sample of 24 children rather than the full sample (*n* = 53). Although this reduced sample size may have limited the statistical power of the engagement analyses, the approach was necessitated by the availability of only two trained observers and the need to comply with the instrument’s recommended observer-to-child ratio. Future studies employing larger teams of trained observers are needed to replicate and extend these findings in larger samples. Another limitation is that the research assistants were not blinded to group allocation. Consequently, assessment bias cannot be completely ruled out, particularly for observational measures such as engagement.

## 5. Conclusions

The most common duration of classroom active break (AB) interventions is approximately 15–20 min [64,65]. However, a typical preschool lesson lasts 40–45 min, making it impractical for an active break to occupy nearly half of the lesson time. Moreover, 20 min corresponds to the duration of a regular break in kindergarten, during which children engage in free play. For this reason, many teachers consider an AB duration of more than 5 min to be unrealistic [18]. For future AB interventions to be both feasible and effective in preschool settings, teachers’ concerns regarding the duration of classroom AB should be taken into account. A unique contribution of this study is the finding that a brief, 5-min music–movement activity is sufficient to improve inhibitory control in preschool children. Furthermore, the higher levels of engagement observed in the intervention group relative to baseline suggest that music–movement activities may have a positive effect on children’s subsequent focus and participation during learning activities. Children’s engagement in the class activities is considered a key factor in improving academic performance [66]. Although the AB group did not show differences in mathematics compared to the sedentary group, the observed improvements in inhibition and engagement suggest that even a brief 5-min AB may facilitate changes in cognitive performance.

## Figures and Tables

**Table 1:** Means and standard deviations of the dependent variables (M ± SD).

	Treatment Group		Control Group	
	Pre	Post	Pre	Post
Flanker test (msec)	1166.71 ± 257.91	963.33 ± 232.70 *	1262.93 ± 269.21	1179.50 ± 308.36
Flanker test (percent %)	65.85 ± 17.37	74.79 ± 18.75	68.15 ± 15.89	74.87 ± 20.56
Corsi block test (number of cubes)	3.24 ± 0.77	3.32 ± 0.74	3.04 ± 0.92	3.14 ± 0.97
Tower of London (level)	3.04 ± 1.09	3.08 ± 1.03	2.96 ± 0.79	2.89 ± 0.68
Number line estimation	3.28 ± 1.24	3.46 ± 1.31	3.48 ± 1.38	3.61 ± 1.39
Numerical magnitude comparison	3.44 ± 1.15	3.79 ± 1.19	3.64 ± 1.18	3.82 ± 1.15
Engagement	3.00 ± 0.85	3.58 ± 0.90 **	3.08 ± 0.90	3.33 ± 0.88
Enjoyment		2.60 ± 0.57		2.68 ± 0.61

* Significant difference between the two groups (*p* < 0.05). ** Significant difference between pre- and post-test.

## Data Availability

The data presented in this study are available on request from the corresponding author.

## Abbreviations

The following abbreviations are used in this manuscript:

AB	Activity Breaks
COP	Child Observation in Preschool
EF	Executive Functions
